# Cyfip1 Regulates SynGAP1 at Hippocampal Synapses

**DOI:** 10.3389/fnsyn.2020.581714

**Published:** 2021-02-05

**Authors:** Abhishek Sahasrabudhe, Fatema Begum, Christopher A. Guevara, Chenel Morrison, Kuangfu Hsiao, Nebojsa Kezunovic, Ozlem Bozdagi-Gunal, Deanna L. Benson

**Affiliations:** ^1^Icahn School of Medicine at Mount Sinai, Nash Family Department of Neuroscience, Friedman Brain Institute, New York, NY, United States; ^2^Graduate School of Biomedical Sciences, New York, NY, United States; ^3^Department of Psychiatry, Rutgers New Jersey Medical School, Newark, NJ, United States

**Keywords:** CYFIP1, SynGAP, AMPA receptors, mGluR, PP2A, Rac

## Abstract

In humans, copy number variations in *CYFIP1* appear to have sweeping physiological and structural consequences in the brain, either producing or altering the severity of intellectual disability, autism, and schizophrenia. Independently, *SynGAP1* haploinsufficiency produces intellectual disability and, frequently, autism. Cyfip1 inhibits protein translation and promotes actin polymerization, and SynGAP1 is a synaptically localized Ras/Rap GAP. While these proteins are clearly distinct, studies investigating their functions in mice have shown that each regulates the maturation of synapses in the hippocampus and haploinsufficiency for either produces an exaggerated form of mGluR-dependent long-term depression, suggesting that some signaling pathways converge. In this study, we examined how *Cyfip1* haploinsufficiency impacts SynGAP1 levels and localization, as well as potential sites for mechanistic interaction in mouse hippocampus. The data show that synaptic, but not total, levels of SynGAP1 in *Cyfip1*^+/–^ mice were abnormally low during early postnatal development and in adults. This may be in response to a shift in the balance of kinases that activate SynGAP1 as levels of Cdk5 were reduced and those of activated CaMKII were maintained in *Cyfip1*^+/–^ mice compared to wild-type mice. Alternatively, this could reflect altered actin dynamics as Rac1 activity in *Cyfip1*^+/–^ hippocampus was boosted significantly compared to wild-type mice, and levels of synaptic F-actin were generally enhanced due in part to an increase in the activity of the WAVE regulatory complex. Decreased synaptic SynGAP1 coupled with a CaMKII-mediated bias toward Rap1 inactivation at synapses is also consistent with increased levels of synaptic GluA2, increased AMPA receptor-mediated responses to stimulation, and increased levels of synaptic mGluR1/5 compared to wild-type mice. Collectively, our data suggest that Cyfip1 regulates SynGAP1 and the two proteins work coordinately at synapses to appropriately direct actin polymerization and GAP activity.

## Introduction

Appropriate levels and regulation of Cyfip1 are important for brain development and function. In humans, either increases or decreases in *CYFIP1* gene dosage are risk factors for intellectual disability, autism, and schizophrenia (Chai et al., 2003; Kirov et al., 2009; van der Zwaag et al., 2010; Leblond et al., 2012; De Rubeis et al., 2014; Kushima et al., 2018), and deletions in chromosome 15 that include *CYFIP1* are associated with increased symptom severity in Prader–Willi and Angelman syndromes (Chai et al., 2003; Butler et al., 2004; Bittel et al., 2006; Sahoo et al., 2006). In rodents, *Cyfip1* manipulation has strong anatomical, cellular, and physiological consequences that overlap mechanistically with cell signaling pathways employed by other genes relevant to intellectual disability, autism, and schizophrenia (Bozdagi et al., 2012; Dominguez-Iturza et al., 2019; Fricano-Kugler et al., 2019; Silva et al., 2019). Such studies suggest that Cyfip1-regulated pathways are part of a nexus of vulnerable developmental events.

An example of this is that mice haploinsufficient for *Cyfip1* show greatly enhanced mGluR1/5-dependent long-term depression (LTD) in the hippocampus that is independent of the usual requirement for protein synthesis (Bozdagi et al., 2012). This phenomenon is strikingly similar to what is observed in mice lacking fragile X mental retardation protein (FMRP; Huber et al., 2002), and the shared dysregulated protein synthesis is consistent with data showing that Cyfip1 and FMRP can bind to one another and act together to repress protein translation (Schenck et al., 2001; Napoli et al., 2008). Interestingly, reduced levels of SynGAP1, a synaptic Ras/Rap GTPase activating protein (Chen et al., 1998; Kim et al., 1998) in humans, can cause a syndromic form of intellectual disability (Holder et al., 1993; Chen et al., 1998; Kim et al., 1998) and also produce enhanced mGluR-dependent, protein synthesis independent LTD in mouse hippocampus (Barnes et al., 2015). The mechanisms by which Cyfip1, FMRP, or SynGAP modifies mGluR signaling are not fully understood, but emerging themes of dysregulated protein synthesis and ERK signaling support the general idea that there are shared, vulnerable pathways (Schenck et al., 2003; Rumbaugh et al., 2006; Carlisle et al., 2008; De Rubeis et al., 2013; Zhao et al., 2013; Pathania et al., 2014; Barnes et al., 2015; Hsiao et al., 2016; Paul et al., 2019). Whether or how SynGAP and Cyfip1 regulatory pathways are related has not been investigated.

In addition to pathways regulating protein synthesis and signaling, SynGAP1 and Cyfip1 may also share pathways regulating F-actin polymerization. Actin cytoskeleton gives dendritic spines their characteristic shape and in excitatory neurons plays a critical role anchoring AMPA receptors (AMPARs) at synapses (Allison et al., 1998; Zhang and Benson, 2000). When Cyfip1 levels are reduced in neurons during development, presynaptic vesicle release probability and terminal size are increased, inhibitory synaptic activity is increased, and postsynaptic dendritic spines fail to develop properly, remaining thin and immature in appearance (De Rubeis et al., 2013; Pathania et al., 2014; Hsiao et al., 2016; Davenport et al., 2019). These actions at synapses are mediated largely by Cyfip1’s participation in the WAVE regulatory complex (WRC), which promotes the generation of branched actin filaments in response to Rac activation and binding (Kunda et al., 2003; Schenck et al., 2003; Steffen et al., 2004; Abekhoukh et al., 2017). Significantly, reduced levels of SynGAP1 enhance Rac activation and have been associated with an increase in dendritic spine size (Vazquez et al., 2004; Carlisle et al., 2008; Clement et al., 2012).

Based on the idea that the actions of Cyfip1 would have mechanistic overlap with those of SynGAP1, we examined how reduced levels of Cyfip1 impacted key measures of synapse function and regulation that are also relevant to SynGAP1. The data show that Cyfip1 regulates the localization and anchoring of SynGAP1, shifting the balance of signaling pathways in a manner that alters baseline levels of AMPAR subunits and mGluR1s in the hippocampus. These data illustrate how modest changes in the level of a single protein can be amplified at synapses.

## Results

### SynGAP Levels Are Abnormally Low in Synaptosome Fractions From *Cyfip1*^+/–^ Mice

**FIGURE 1:**
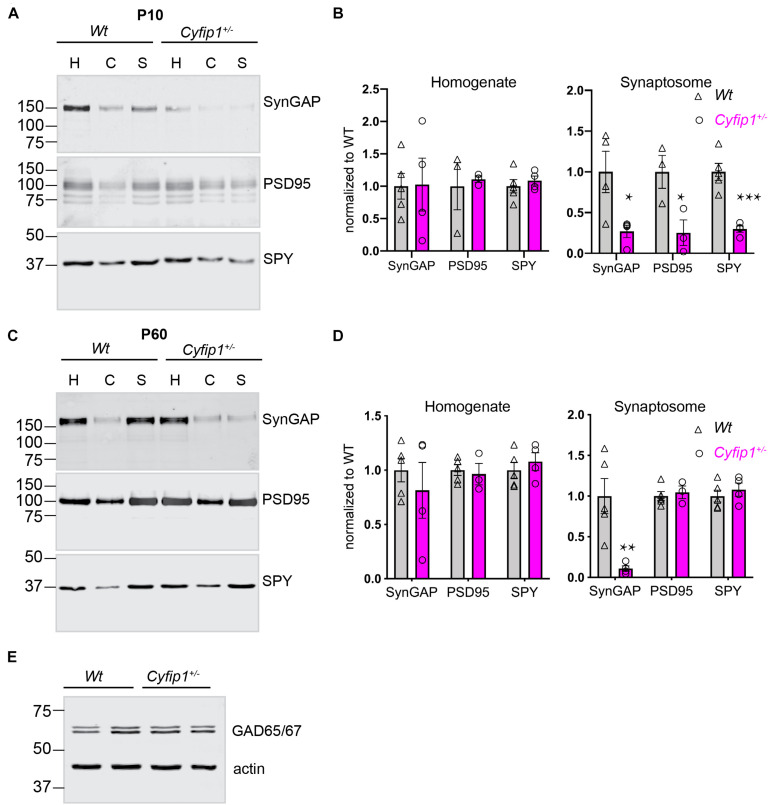
Compositional differences in developing and mature synaptic fractions from *Cyfip1*^+/–^ mice. P10 **(A)** and P60 **(C)** hippocampi fractionated on 10% gels, 25 μg per lane and blotted for the antibodies indicated. H, homogenate; C, cytoplasmic fraction; S, synaptosome fraction. Mean intensities are shown at the right in **(B)** (P10) and **(D)** (P60). *p ≤ 0.05; **p = 0.014; ***p = 0.008; unpaired *t*-tests with Welch’s correction (*n* = 5 *Wt* and 4 Cyfip1 except for P60 PSD95 where *n* = 3 Cyfip1). Levels of GAD65/67 in hippocampal total lysates (statistics in the text) **(E)**.

In hippocampal glutamatergic terminals of Cyfip1 haploinsufficient (*Cyfip1*^+/–^) mice, presynaptic vesicle size and release probability are increased during development, but the effect is transient and recovers by postnatal day 21 (P21). At P21 and later, amplitudes of excitatory currents appear normal, but there is an increased density of thin dendritic spines, and activity-dependent AMPAR recycling is disrupted (De Rubeis et al., 2013; Pathania et al., 2014; Hsiao et al., 2016). These data suggest that there may be abnormalities in the protein scaffold supporting the structure of synapses in *Cyfip1*^+/–^ mice. To examine this, we compared the distribution and levels of canonical pre- and post-synaptic proteins (synaptophysin and PSD95, respectively) and SynGAP1, which is enriched postsynaptically, in total homogenates and synaptosome fractions prepared from *Wt* and *Cyfip1*^+/–^ mice at two ages, P10 and P60. At P10, most synapses are nascent, there are few dendritic spines, and synaptosome fractions contain growth cones, as well as synapses. The data from P10 mice show that the accumulation of all three proteins in synaptosomal fractions is reduced in *Cyfip1*^+/–^ fractions compared with *Wt* (Figures 1A,B), whereas levels in total homogenates are similar between genotypes. On its surface, these findings suggest that there may be fewer synapses in *Cyfip1*^+/–^ hippocampus, but this is unlikely based on previous experiments, which showed that at this age, the density of immunolabeled presynaptic terminal puncta in tissue sections from hippocampal CA1 was similar in *Cyfip1*^+/–^ and *Wt*, and that miniature excitatory postsynaptic current (mEPSC) frequency in CA1 stratum radiatum was actually increased in *Cyfip1*^+/–^ mice at P10 compared with *Wt* (Hsiao et al., 2016). Thus, reduced levels of pre- and post-synaptic proteins in synaptic fractions from *Cyfip1*^+/–^ mice probably reflect differences in protein–protein or protein–cytoskeletal interactions that impact how proteins separate into particular biochemical fractions.

At P60, the distribution of synaptophysin and PSD95 in both total homogenates and synaptosome fractions is similar between *Cyfip1*^+/–^ and *Wt* mice. Levels of SynGAP1 in total homogenates are also similar between *Cyfip1*^+/–^ and *Wt*, but SynGAP1 levels in *Cyfip1*^+/–^ synaptosome fractions remain abnormally low and are similar to what is observed at P10 (Figures 1C,D).

Because Cyfip1 can regulate local protein synthesis as part of a complex with FMRP (Napoli et al., 2008), we used translating ribosome affinity purification (TRAP) to address whether SynGAP1 translation was suppressed in *Cyfip1*^+/–^ mice. However, on ribosomes purified from CA1, there was no significant decrease in levels of SynGAP1 transcripts in *Cyfip1*^+/–^ compared with *Wt* mice (log fold change = −0.067, *p* = 0.78, *n* = 3 *Wt* and 4 *Cyfip1*^+/–^ mice). As these data are consistent with the absence of SynGAP1 regulation in CA1 from *Fmr1*^–/y^ mice, assessed either by TRAP (log fold change = −0.075, *p* = 0.61, *n* = 3 *Wt* and 3 *Fmr1*^–/y^) (Thomson et al., 2017) or by RiboTag (log fold change = −0.15, *p* = 0.45, *n* = 6 *Wt* and 6 *Fmr1*^–/y^) (Ceolin et al., 2017), it is unlikely that Cyfip1 and FMRP repress SynGAP1 translation.

The maturation of GABAergic synapses has been shown to be altered in the neurons expressing increased levels of Cyfip1 (Davenport et al., 2019). Based on this, we compared levels of GAD65/67 by Western blot in *Cyfip1*^+/–^ and *Wt* mice, but we observed no differences in levels (Figure 1E; GAD/actin; Mann–Whitney test, *p* = 0.8), similar to what has been reported for Gephyrin in mice having a conditional deletion of Cyfip1 (Davenport et al., 2019).

### SynGAP Puncta Are Reduced at PSDs *in situ*

**FIGURE 2:**
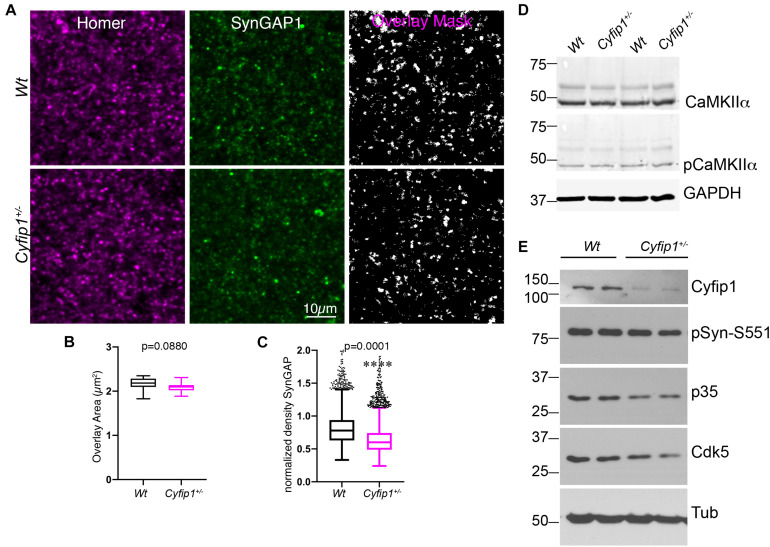
Reduced synaptic SynGAP and Cdk5 activity. Area CA1 in *Wt* and *Cyfip1*^+/–^ mice was imaged at high magnification in sections immunolabeled for SynGAP1 [green; **(A)**] and Homer1b/c [red; **(A)**], analyzed using ImageJ [overlay mask in panel **(A)**]. Box plots show the mean area of overlap **(B)** and the integrated intensity of SynGAP puncta **(C)** in *Wt* and *Cyfip1*^+/–^ CA1. Data were compared using unpaired *t*-test, *p* = 0.0880 **(B)** and *p* = 0.0001 **(C)**. **(D)** Western blot for total and phosphorylated CaMKIIα (comparison values are in the text). **(E)** Western blots for Cdk5, its activator, p35, and a substrate, p-Synapsin-Ser 551 in *Wt* and *Cyfip1*^+/–^ mutant mouse hippocampi (comparison values are in the text).

Biochemical data support that levels of SynGAP1 associated with postsynaptic densities (PSDs)s are tightly regulated (Gamache et al., 2020; Zhang et al., 2020). To confirm that the decreased synaptosomal levels of SynGAP1 reflect decreased association with PSDs, we determined the percentage of immunolabeled SynGAP1 puncta that were associated with putative postsynaptic sites identified by Homer (a pan-glutamatergic PSD marker) in tissue sections from CA1 stratum radiatum. In high magnification confocal images acquired from the hippocampus of *Wt* or *Cyfip1*^+/–^ mice, we applied a multiplication-based analysis strategy in ImageJ to compare Homer/SynGAP1 overlapping puncta (Figure 2A). For sites having both labels, the extent of overlap was similar between the two genotypes (Figure 2B), but consistent with the Western blot data, SynGAP1 intensity at sites delineated by Homer was reduced significantly in *Cyfip1*^+/–^ mutants compared with *Wt* (Figure 2C). *Cyfip1*^+/–^ and *Wt* hippocampi had similar densities of Homer clusters, supporting equal densities of postsynaptic structures, as expected (*t*-test, *p* = 0.9257, *n* = 3). The overall density of SynGAP1 puncta did not differ between genotypes either (*t*-test, *p* = 0.2634, *n* = 3). These data support reduced SynGAP1 anchoring at synapses in *Cyfip1*^+/–^ mice.

### Cyfip1 Haploinsufficiency Decreases Cdk5 Activity

Mechanisms supporting SynGAP1 recruitment and retention are differentially modulated downstream of CaMKII- or Cdk5-mediated phosphorylation (Walkup et al., 2015). Based on this, we asked whether either kinase showed altered activation in *Cyfip1*^+/–^ mice. In Western blots, the data show no differences between genotypes in levels of total and phosphorylated (activated) CaMKIIα (Figure 2D; pCKIIα/totCKIIα; *t*-test, *p* = 0.2186). In contrast, total levels of Cdk5, its activator p35, and Synapsin I phosphorylation at S551, a Cdk5 site (Matsubara et al., 1996), were consistently reduced in *Cyfip1*^+/–^ mice compared with *Wt* (Figure 2E; Cdk5; *t*-tests, *p* = 0.04; p35, *p* = 0.008; pSyn, *p* = 0.03; Cyfip1, *p* = 0.0003). Based on previous work assessing the impact of SynGAP1 phosphorylation on its GAP activity (Walkup et al., 2015), these data suggest that with decreased levels of Cyfip1, SynGAP1 activity would be biased toward Rap.

### Glutamatergic Activity and GluA2 Levels Are Increased in *Cyfip1*^+/–^ Mice

**FIGURE 3:**
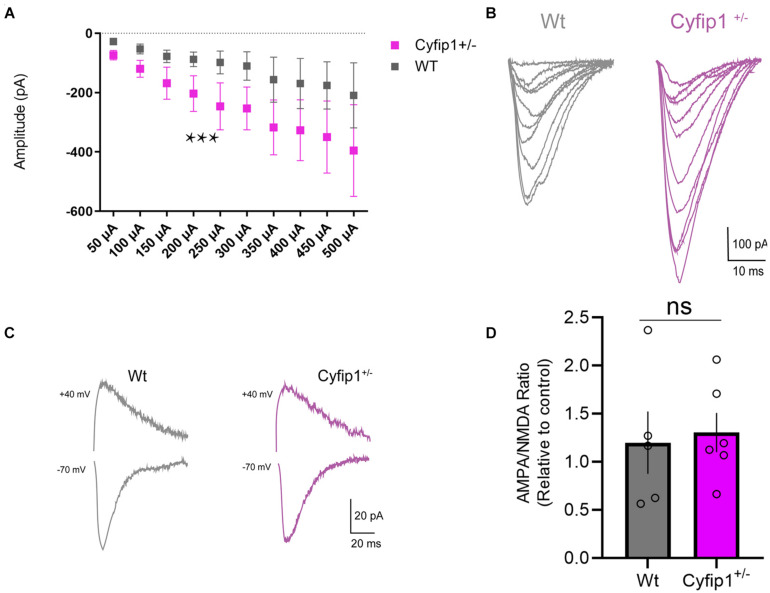
AMPA receptor regulation in *Cyfip1*^+/–^ mice. Whole cell recordings **(A)** show increased AMPA receptor responses in CA1 in response to Schaeffer collateral stimulation (slope *Wt* = 0.327; slope *Cyfip1*^+/–^ = 0.683; *p* = 0.0003, mixed effects analysis, *n* = 4 each). Individual traces **(B)** from the data shown in **(A)**. Representative traces of AMPA and NMDA currents **(C)** from *Wt* and *Cyfip1*^+/–^ mice. Quantification of AMPA:NMDA ratios **(D)** shows no significant differences between the two genotypes. Groups were compared using unpaired *t*-test, *p* = 0.7832.

Our data and those of others support that spontaneous EPSC frequency and amplitude are similar in adult *Cyfip1*^+/–^ and *Wt* mice (Hsiao et al., 2016; Davenport et al., 2019). However, decreased levels of SynGAP or reduced Cdk5 activity would be expected to enhance AMPA responses (Kim et al., 2003; Walkup et al., 2015; Jeyabalan and Clement, 2016). Whole cell recordings of CA1 neurons in hippocampal slices were used to assess AMPAR-mediated currents in response to a range of stimulation intensities. The data show that amplitudes of AMPAR-mediated responses were consistently greater in *Cyfip1*^+/–^ relative to *Wt* neurons over a range of depolarizing current steps (Figures 3A,B). However, the increase in AMPAR responses did not translate into increased AMPA:NMDA ratios. When we recorded evoked currents at −70 and +40 mV (Figure 3C), there was no difference between *Cyfip1*^+/–^ and *Wt* mice in AMPA:NMDA ratios (Figure 3D).

**FIGURE 4:**
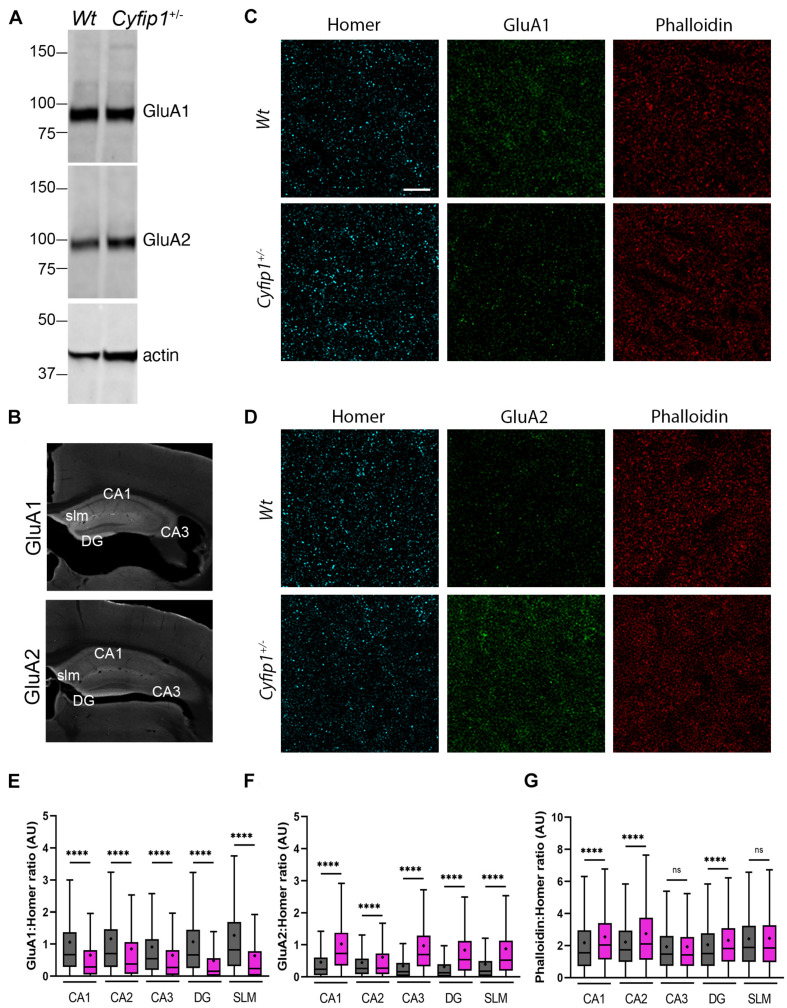
AMPA receptor and F-actin regulation in *Cyfip1*^+/–^ mice. Western blot **(A)** suggests that GluA1 and GluA2 levels are similar in *Wt* and *Cyfip1*^+/–^ hippocampal lysates. Representative low magnification images **(B)** of GluA1 and GluA2 immunostained hippocampal sections highlighting the regions in which dendritic zones were evaluated quantitatively. Confocal images **(C,D)** of synaptic puncta immunostaining for Homer1 (cyan), GluA1, GluA2 (green), or Phalloidin (red). Homer puncta were used to identify the potential synapses and were used as masks to measure grayscale intensities for Homer, GluA1, GluA2, and Phalloidin. Homer intensities were used to normalize the corresponding GluA1, GluA2, and Phalloidin intensities **(E–G)**. Groups were compared using one-way ANOVA (Kruskal–Wallis test), followed by Dunn’s multiple comparison, ^****^p < 0.0001 (scale bar = 10 μm).

We next asked whether the change in AMPA responses reflected increased levels of particular AMPAR subunits. TRAP data showed no significant differences in levels of any of the mRNA transcripts encoding AMPAR (*Gria1-4*) in *Cyfip1*^+/–^ compared with *Wt* mice (log fold change range, −0.017 to −0.24, *p* range, 0.17–0.92, *n* = 3 *Wt* and 4 *Cyfip1*^+/–^ mice). Western blots of hippocampal tissue lysates also showed no obvious differences in GluA1 or GluA2 levels between *Wt* and *Cyfip1*^+/–^ mutants (Figure 4A). However, changes in regional or synaptic distribution could be masked in whole hippocampal lysates. To address this possibility, hippocampal sections were labeled for Homer, GluA1 or GluA2, and phalloidin to label F-actin, and then using Homer1 puncta to identify synaptic regions, labeling intensity was assessed at high magnification in regions sampled from CA1 and CA3 stratum radiatum, dentate gyrus molecular layer, and stratum lucidum (Figures 4B–D). The data show that GluA1 levels were consistently lower in *Cyfip1*^+/–^ than in *Wt* mice, and that conversely, GluA2 levels were increased (Figures 4E,F). F-actin levels were unchanged in CA3 and SLM, but were significantly elevated in CA1, CA2, and dentate gyrus (Figure 4G). The data suggest that levels of Cyfip1 regulate GluA subunit composition at synapses.

### Synaptic Levels of mGluR1/5 Are Enriched in CA Fields

**FIGURE 5:**
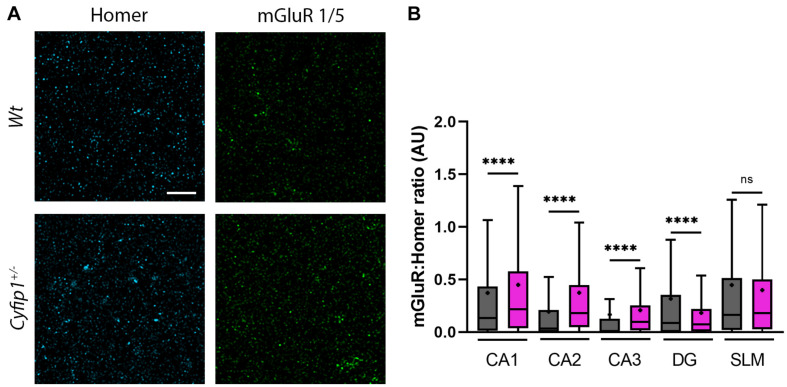
mGluR and F-actin regulation in *Cyfip1*^+/–^ mice. Confocal images **(A)** of synaptic puncta immunostaining for Homer1 (cyan) and mGluR1/5 (green). Homer puncta were used to identify the potential synapses and were used as masks to measure grayscale intensities for Homer and mGluR1/5. Homer intensities were used to normalize the corresponding mGluR intensities **(B)**. Groups were compared using one-way ANOVA (Kruskal–Wallis test), followed by Dunn’s multiple comparison, ^****^p < 0.0001 (scale bar = 10 μm).

Since mGluR5-mediated function is dysregulated at Schaeffer collateral synapses in mice haploinsufficient for either *Cyfip1* or *SynGAP1* (Bozdagi et al., 2012; Barnes et al., 2015), we asked whether spatial relationships between immunolabeled mGluR1/5 and Homer, its PSD binding partner, were different in *Cyfip1*^+/–^ mice. Using an approach similar to that for GluAs, Homer puncta were used to define synaptic regions of interest in which Homer and mGluR1/5 labeling intensity were assessed. The intensity of mGluR1/5 within Homer domains increased significantly in CA1, CA2, and CA3, but there were no changes observed in SLM (Figure 5). These data suggest that enhanced levels of synaptic mGluR1/5 may contribute to the exaggerated mGluR-dependent LTD observed in *Cyfip1*^+/–^ mice.

**FIGURE 6:**
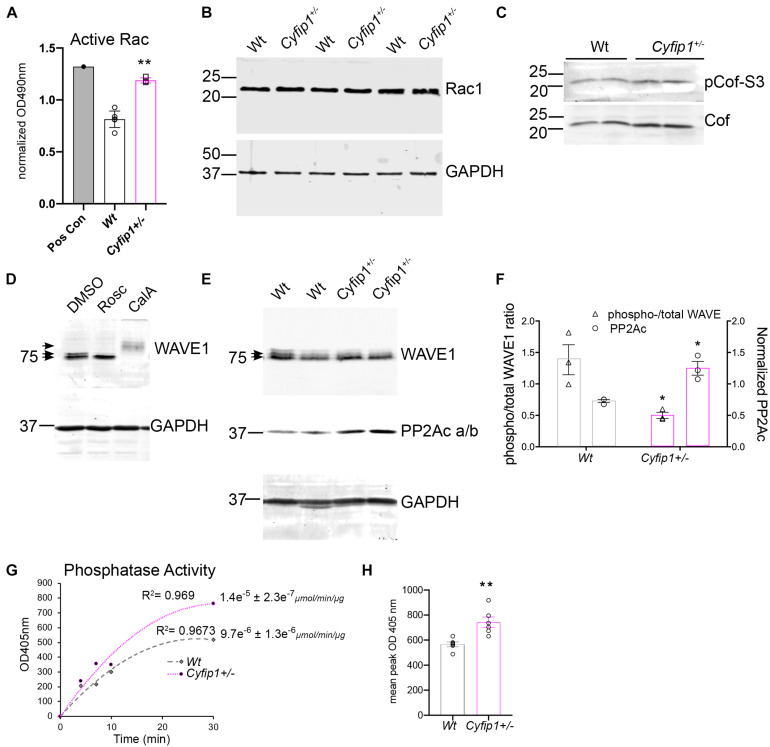
Cyfip1 haploinsufficiency increases Rac1 activity. **(A)** Bar graph shows the results from ELISA for Rac activity (unpaired *t*-test, **p = 0.002; *n* = 4 *Cyfip1*^+/–^ and 6 *Wt*). **(B)** Western blot shows Rac1 levels in total hippocampal lysates from the same preparations used for the data in (**A**; statistics in the text). **(C)** Western blots for total and phosphorylated cofilin in lysates from the hippocampus (statistics in the text). **(D)** Western blots of WAVE1 from cell lysates in response to treatments indicated. **(E)** Blots of WAVE1 and PP2Ac a/b in hippocampal lysates from *Wt* and *Cyfip1*^+/–^ mice. **(F)** Graph of data in **(E)** showing the ratio of upper “phospho” to total WAVE1 (left *y* axis) and PP2Ac intensity (right *y* axis) (unpaired *t*-tests; pWAVE/WAVE1; *p = 0.002; PP2Ac, *p = 0.04, *n* = 3 each). **(G)** Phosphatase activity assay and **(H)** mean comparison of max OD at 405 nm, **p = 0.003, *n* = 6 each.

There are a variety of potential sources for increasing synaptic actin polymerization. Based on previous work in the laboratory, we used an ELISA-based activity assay to measure Rac1 activity in synaptic fractions isolated from *Wt* and *Cyfip1*^+/–^ hippocampi. The data show that Rac1 activity was increased significantly in the *Cyfip1*^+/–^ fractions compared with *Wt* (Figure 6A). This occurred in the absence of any noticeable change in total levels of Rac1 in immunoblots (Figure 6B, *t*-test; *p* = 56; *n* ≥ 4). Active Rac can promote actin assembly by a pathway that decreases cofilin activity, but we observed no significant differences in levels of cofilin phosphorylation (Figure 6C; Mann–Whitney test, pCof/totCof; *p* = 0.19; *n* ≥ 4). Alternatively, active Rac also promotes WRC activity. As an essential subunit, decreased Cyfip1 levels serve to reduce levels of WRC, so we asked whether the remaining WRC is more active. Since WAVE1 activity is negatively regulated by phosphorylation (Kim et al., 2006), we first confirmed that we could detect phosphorylated WAVE1 by Western blot. A brief treatment with Cdk5 inhibitor, roscovitine, facilitated WAVE1 mobility and yielded a single, lower MW band. In contrast, treatment with calyculin A, a PP2A inhibitor, produced a super shift in WAVE1 bands (Figure 6D). In lysates from *Cyfip1*^+/–^ hippocampus, high MW bands were reduced compared with *Wt*, consistent with decreased WAVE1 phosphorylation (Figures 6E,F). We did not detect an increase in PP2A isoforms in our TRAP data, but in previous work, the mRNA encoding the catalytic subunit of the Ser/Thr phosphatase 2A (PP2Acβ) was identified as a regulatory target of FMRP and Cyfip1 (Castets et al., 2005; Darnell et al., 2011). Based on this, we probed for PP2Acα/β subunits by Western blot. The data show a negative correlation between levels of PP2Ac and levels of WAVE1 phosphorylation (Figures 6E,F). We further examined this difference by assessing levels of phosphatase activity directly using a p-nitrophenylphosphate – a pan phosphatase chromogenic substrate. The data show increased phosphatase activity in *Cyfip1*^+/–^ hippocampus compared with *Wt* (Figures 6G,H). Collectively, these data support that F-actin levels may be increased in part by the compensatory activation of WRC.

## Discussion

**FIGURE 7:**
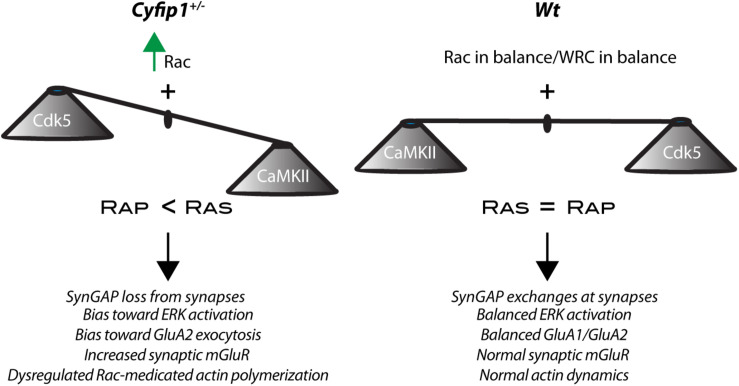
Cyfip1 haploinsufficiency alters the balance of activity. Diagram places the results from this study into a larger framework outlined in the section “Discussion.”

Recent research shows that SynGAP1 homo-trimers *in vitro* can bind multiple copies of PSD95 provoking a phase separation of the complex (Zeng et al., 2016). While implications of such interactions are not understood, the data suggest that PSDs lacking SynGAP1 would have an altered organization. To a similar end, but by a different mechanism, a separate study has shown data suggesting that decreased levels of synaptic SynGAP1 can open “slots” in PSD95, permitting interactions with alternate partners and promoting a change in PSD composition (Walkup et al., 2016; Lautz et al., 2018). The data presented here support the idea that synapse composition is altered in the hippocampus of *Cyfip1*^+/–^ mice by having reduced levels of SynGAP1 and GluA1 at synapses and enhanced levels of mGluR1/5, GluA2, and F-actin compared with *Wt*. Collectively, the data suggest that changes in composition are driven in part by a shift in the balance of SynGAP1’s location and its activity toward Ras and Rap at synaptic and non-synaptic sites (Figure 7).

Our data support a model in which Cyfip1 plays a role anchoring SynGAP1 to the PSD, since synaptic, but not total, levels of SynGAP1 were diminished in *Cyfip1*^+/–^ mice. The loss of SynGAP1 from synaptosomal fractions is stark and nearly complete (Figure 1), and while this could be due to a deficiency in either the targeting or anchoring of SynGAP1, the more modestly, but still significantly, reduced overlap between SynGAP1 and Homer seen in intact immunolabeled preparations (Figure 2) better supports the idea that anchoring or short range interactions are altered. This difference in SynGAP1 localization is likely to be mediated by changes in the composition of actin cytoskeleton. Our data show increased levels of Homer-associated F-actin in CA1, CA2, and dentate gyrus (Figure 4), and that this may reflect a compensatory increase in WRC activity (Figure 6). Levels of active Rac, the upstream activator for WRC, were increased and are consistent with previous work showing that Rac inhibition rescued deficits in synapse function in *Cyfip1*^+/–^ mice (Hsiao et al., 2016) and phosphorylation of WAVE1, which negatively regulates its activity (Kim et al., 2006), was decreased. At the same time, there was no change in cofilin phosphorylation (and inactivation), which downstream of Rac activation can enhance F-actin polymerization (Yang et al., 1998; Chen et al., 2010). No matter the pathway, however, any increase in F-actin appears to be insufficient to generate normal synapse structure as it fails to corral or support appropriate levels of SynGAP1 trafficking and anchoring (Figure 1), and previous work suggests that it also fails to support the generation of normal spine shape (De Rubeis et al., 2013; Pathania et al., 2014).

AMPA receptor levels typically scale with spine size (Kopec et al., 2007), but this coordinated regulation appears to be altered when Cyfip1 levels are reduced. Spine size is reduced in *Cyfip1*^+/–^ neurons (De Rubeis et al., 2013; Pathania et al., 2014), and our data show that CA1 synapses have increased AMPAR responses. The increased AMPA current may reflect enhanced levels of GluA2, but GluA1 levels are reduced, and AMPA/NMDA ratios remain similar to *Wt* neurons (Figures 3, 4). Changes in AMPAR levels and composition could lie downstream of decreased levels of Cdk5 activity (Figure 6). The decreased Cdk5 and p35 levels that we observe in *Cyfip1*^+/–^ hippocampus (Figure 2) would be expected to bias any remaining SynGAP activity toward Rap inactivation (Walkup et al., 2015), an action that would be expected to favor GluA2 exocytosis (Huang et al., 2004; Fu et al., 2007). This effect could help to counter the impact of longer and thinner spines, since hippocampal LTD induced by any of a variety of protocols in *Cyfip1*^+/–^ mice is similar to that evoked in *Wt* (Bozdagi et al., 2012). Enhanced GluA2 levels may also generate a favorable state for mGluR-dependent LTD (Waung et al., 2008; Pick et al., 2017).

mGluR-dependent LTD is exaggerated in *Cyfip1*^+/–^ mice, independent of the normal requirement for protein synthesis (Bozdagi et al., 2012), and similar to what is observed in mice lacking FMRP (Huber et al., 2002) or having reduced levels of SynGAP1 (Barnes et al., 2015). This is thought to be in part due to dysregulated protein translation, which appears to be de-repressed in all three mouse models through partially overlapping mechanisms: Cyfip1 and FMRP are binding partners that work together to suppress a fraction of protein translation at mRNA start sites binding FMRP, FMRP additionally prevents mRNA translation by binding to other sites (Schenck et al., 2001; Napoli et al., 2008; Darnell et al., 2011), and FMRP levels are reduced in mice haploinsufficient for SynGAP1 (Darnell et al., 2011; Paul et al., 2019). It has been challenging, however, to draw straight lines between the mRNAs targeted by Cyfip1 and FMRP and functional outcomes observed (Ceolin et al., 2017; Thomson et al., 2017; Das Sharma et al., 2019, this paper), and it is significant that enhanced Ras-ERK1/2 signaling has emerged as a target that is common to *SynGAP1*^+/–^ and *Fmr1*^–/y^ mutant mice (Rumbaugh et al., 2006; Osterweil et al., 2010; Barnes et al., 2015). Our data from *Cyfip1*^+/–^ mice are consistent with this idea in that decreased levels of SynGAP1 at synapses and decreased Cdk5 signaling would be expected to generate a bias toward Ras signaling. This imbalance could also contribute to the increased levels of mGluR1/5 observed at Homer-labeled sites in CA fields, providing an additional source for enhanced mGluR-dependent signaling in *Cyfip1*^+/–^ mice. Increased mGluR levels may also be related to what has been observed in mice lacking FMRP where mGluRs are triton-extractable (Giuffrida et al., 2005) and more mobile than *Wt* mice (Aloisi et al., 2017). Additionally, it is significant that enhanced mGluR5 levels were not observed in SLM in the *Cyfip1*^+/–^ mice, a hippocampal region that does not express SynGAP1 (Porter et al., 2005) and fails to undergo mGluR-dependent LTD (Fitzjohn et al., 2016).

Together our data reveal that Cyfip1 regulates the synaptic expression of AMPARs, SynGAP1 and mGluRs. Reduced levels of Cyfip1 enhance Rac signaling, and downstream of SynGAP1, alter the balance between Ras and Rap signaling, ultimately shifting the range and flexibility of synapse responses (Figure 7).

## Materials and Methods

### Mice

*Cyfip1* haploinsufficient mice were bred as heterozygotes to generate *Cyfip1*^+/–^ mice and *Wt* littermates. Unless otherwise noted, mice were ∼2 months old and included both males and females. Sex was noted and parsed for analyses, but no differences or even trends toward differences were observed, and males and females were grouped. For biochemical and electrophysiological experiments, mice were deeply anesthetized with isoflurane and then decapitated; brains were removed and dissected. For immunolabeling, mice were deeply anesthetized with ketamine and xylazine, and brains were fixed by transcardial perfusion with 4% paraformaldehyde (PFA) in phosphate buffered saline (PBS), pH 7.3. Brains were removed, postfixed overnight in the same fixative, and then placed in 4% sucrose in PBS. Tissue sections were acquired through the dorsal hippocampus using a freezing microtome at a setting of 30 μm.

### Antibodies

Primary antibodies included: mouse anti-GluA1 (NeuroMAB; 1:10 IHC), rabbit anti-GluA1 (Cell Signaling Technologies; 13185, 1:1,000), mouse anti-GluA2 (NeuroMAB; 1:10 IHC), mouse anti-GluA2 (MAB1189; 1:500), chicken anti-Homer (SySy; 160006, 1:500), mouse anti-SynGAP (Thermo Fisher Scientific; PA1-046, 1:500 IHC, 1:5,000 WB), mouse anti-PSD95 (Thermo Fisher; MA1-045, 1:1,000 WB), rabbit anti-Synaptophysin (PA1-1043, 1:5,000 WB), mouse anti-Cdk5 (Invitrogen; AHZ0492, 1:500), mouse anti-WAVE1 (mAb K91/361; NeuroMab), goat anti-PPA2c (Santa Cruz; sc-6110), rabbit anti-CaMKIIα (Abcam; EP1829Y, 1:1,000), rabbit-anti-p-Synapsin1-Ser 551 (Abcam; Ab32532), rabbit anti-p35 (Cell Signaling; 2680, 1:250), mouse anti-GAPDH (Millipore; MAB374, 1:4,000), and Phalloidin Alexa 647 (Thermo Fisher Scientific; A22287, 1:200). Secondary antibodies included: donkey anti-mouse Alexa 488 (Thermo Fisher Scientific; R37114, 1:200), donkey anti-mouse Dy 488 (Abcam; 96875, 1:500), donkey anti-chicken Alexa 488 (Jackson ImmunoResearch; 703-545-155), and LI-COR (anti-mouse, anti-goat, and anti-rabbit IRDye 680 and 800; 1:10,000).

### Immunohistochemistry

Tissue sections were permeabilized with 0.50% Triton X-100 in PBS for 15 min, washed six times for 10 min in PBS, and blocked in 5% normal donkey serum (NDS) for 1 h and 30 min, shaking at room temperature (RT). After removing the NDS blocking buffer, tissue sections were incubated with primary antibodies at 4°C for three nights on a shaker (primary antibodies were diluted in 2% NDS and 0.1% Triton X-100 in PBS). After three nights of incubation, brain sections were washed in PBS, six times for 10 min at RT on a shaker. Secondary antibodies were diluted in 2% NDS and 0.1% Triton X-100 in PBS, and incubation of tissue sections included shaking for 1 h at RT, shielded from light. After incubation was completed, six 10-min washes at RT were performed. Next, tissue sections were mounted on Superfrost Plus slides using Vectashield that included approximately 5 ng/ml of DAPI for widefield and with Prolong Diamond (Molecular Probes) for confocal microscope preparations. Slides were sealed using nail polish and dried overnight in the dark.

### Image Acquisition and Analysis

To compare receptor distribution and immunolabeling intensity in hippocampal fields, images were captured on a Leica DMi8 widefield microscope at 10× magnification, with an exposure time of 500 ms and no binning. Images were exported, stitched, and analyzed using ImageJ. Using the rectangle tool, five boxes, ∼48 μm^2^, were overlaid in the CA1, CA2, CA3, SLM, and DG regions. Mean intensity levels were measured and recorded. To analyze the overall distribution and overlap of SynGAP1 and Homer-labeled puncta, images were captured on a Leica 780 LSM confocal microscope using a 63× 1.4 NA objective. In ImageJ, a mask of the Homer and SynGAP1 puncta was created and multiplied to assess overlap. To assess levels of GluA1, GluA2, mGluR1/5, and F-actin at Homer-labeled sites, labeled sections were imaged on a Leica SP8 STED using a 100× objective (1.5 NA) and deconvolved using Huygens (SVI), and intensity was measured within a region of interest (ROI) defined by Homer1b/c labeling using ImageJ. GluA1, GluA2, and Phalloidin signals were normalized to the respective Homer signals in individual images. Data were exported to Excel, and groups were compared and plotted using Prism (GraphPad).

### Western Blot

The hippocampus, cortex, or cortical cells grown in culture were solubilized in ice cold RIPA or Syn-PER (Thermo Fisher Scientific) lysis buffer containing protease (Roche) and phosphatase (Life Technologies) inhibitors as detailed previously (Matikainen-Ankney et al., 2016). Then, 25–75 μg of each protein were loaded per lane on 8–10% SDS gels, blotted, labeled with antibodies indicated, and visualized using LI-COR Odyssey. Band intensities were measured using the Gel Analyzer tool in ImageJ. GluA1 and GluA2 Western blots were carried out with the assistance of Shakti BioResearch (Woodbridge, CT, United States).

### Electrophysiology

Whole cell recordings were carried out in acute, coronal hippocampal slices (350 μm) from 4 Wt and 4 *Cyfip1*^+/–^ adult (∼P70) male mice. Slices were cut on a Leica VT1000 vibratome in ice cold aCSF (in mM: 233.7 sucrose, 26 NaHCO_3_, 3 KCl, 8 MgCl_2_, 0.5 CaCl_2_, 20 glucose, and 0.4 ascorbic acid) after which they were allowed to equilibrate in oxygenated recording aCSF (in mM: 117 NaCl, 4.7 KCl, 1.2 MgSO_4_, 2.5 CaCl_2_, 1.2 NaH_2_PO_4_, 24.9 NaHCO_3_, and 11.5 glucose) for 1 h at RT. The neurons were visualized using an upright epifluorescence microscope (BX50WI; Olympus) with 40× water immersion lens and IR-1000 infrared CCD monochrome video camera (DAGE MTI). Whole cell recordings were performed with glass micropipettes filled with high potassium intracellular solution containing (in mM): 124 K-gluconate, 10 HEPES, 10 phosphocreatine di(tris), 0.2 EGTA, 4 Mg_2_ATP, and 0.3 Na_2_GTP. Recordings were made at 31°C in an immersion chamber containing gabazine (GBZ, 10 μM) and APV (40 μM). All AMPA responses were recorded in voltage-clamp mode using a MultiClamp 700B amplifier (Molecular Devices). Analog signals were low-pass filtered at 2 kHz and digitized at 5 kHz with the use of a Digidata 1440A. Gigaseal and further access to the intracellular neuronal compartment were achieved in voltage-clamp mode, with the holding potential set at −70 mV. Soon after rupturing the membrane, the intracellular neuronal fluid reached equilibrium with the pipette solution without significant changes in either series resistance or membrane capacitance values. Membrane voltage was kept at −70 mV though all our voltage-clamp experiments. The input–output (I–O) relationships were measured for AMPAR current amplitudes elicited by stimulating currents with increasing intensity (50–500 μA; 5 stimuli per step). Off-line analysis was performed and analyzed with pClamp10 software (Molecular Devices).

To capture AMPA/NMDA ratios, electrode internal solution consisted of (in mM): 120 Cs-methanesulfonate, 10 HEPES, 0.5 EGTA, 8 NaCl, 5 TEA-CL, 4 Mg-ATP, 0.4 NaGTP, and 10 phosphocreatine. All responses were evoked at 0.1 Hz. AMPA/NMDA ratio was calculated as the peak EPSC value at −70 mV, a timepoint where NMDA response is negligible, divided by the peak response at 40 mV 100 ms after current onset, which is the timepoint where the current response is predominantly NMDA current (Arruda-Carvalho and Clem, 2014). Final ratios were normalized relative to *Wt*.

### Activity Assays

Rac1 activity was measured in dissected hippocampi (P21) from 6 *Wt* mice and 4 *Cyfip1*^+/–^ mice using a Rac1 G-ELISA activation assay kit (Cytoskeleton) according to the manufacturer’s protocol. Absorbance was read at 490 nm using a VICTOR X 4 plate reader (Perkin Elmer). Total levels of Rac1 were examined in samples separated using 15% SDS PAGE, blotted with mouse anti-Rac1 (Cytoskeleton) and rabbit anti-GAPDH (Cell Signaling), and visualized using LICOR. Para-nitrophenylphosphate (pNPP) A phosphatase assay kit (BioAssay Systems) was used to measure phosphatase activity in cortical brain lysates prepared from 2- to 3-weeks-old *Cyfip1*^+/–^ or *Wt* mice (three each) in the absence of phosphatase inhibitors and then mixed with pNPP. Phosphatase activity in the lysates dephosphorylates pNPP and produces para-nitrophenol, which exhibits a strong absorption at 405 nm, which was measured on a plate reader. Activity was calculated according to Lambert–Beer’s law as follows: A = E_405_ ⋅ *V*_*total*_/*t* ⋅ *ε* ⋅ *d* ⋅ *m*_*enzyme*_). As a control, phosphatases in the sample were neutralized by adding Ser/Thr phosphatase inhibitor cocktail 3 (Sigma; P0044) to the pNPP reaction. To detect WAVE1 phosphorylation, cultured, 4-week-old cortical neurons were treated for 45 min with either roscovitine (50 nM; Sigma), calyculin (25 nM; Calbiochem), or vehicle (DMSO).

### TRAP

Three *Wt* and 4 *Cyfip1*^+/–^ male mice were injected with pAAV-FLEX-EGFPL10a and pENN.AAV.CaMKII 0.4.Cre.SV40 in CA1. The site and extent of expression (through about 2/3 of the dorsal hippocampus) were confirmed in pilot studies. The hippocampus was dissected, and the GFP expression was used to pull down ribosomes as described (Heiman et al., 2008). Associated mRNAs were used to generate cDNA. Sequencing and analysis were conducted by GENEWIZ.

### Analysis and Statistics

Genotypes were compared using unpaired *t*-test (unless otherwise mentioned) or for multiple comparisons, by using one-way or two-way ANOVA, or a mixed effects model when appropriate. For all multiple comparisons, *post-hoc* tests were used to identify the source/s of differences. Data were compared, graphed, and plotted using Prism (GraphPad). Numbers and statistical values are provided in the graphs and figure legends, or in the text, when relevant.

## Data Availability Statement

The raw data supporting the conclusions of this article will be made available by the authors, without undue reservation.

## Ethics Statement

The animal study was reviewed and approved by Institutional Animal Care and Use Committee, Icahn School of Medicine at Mount Sinai.

## Author Contributions

AS, KH, OB-G, and DB designed the study. AS, CG, KH, FB, CM, and NK conducted the experiments. AS, CG, KH, FB, CM, and DB analyzed the experiments. AS and DB wrote the manuscript, and all the other authors provided feedback and edits. All authors contributed to the article and approved the submitted version.

## Conflict of Interest

The authors declare that the research was conducted in the absence of any commercial or financial relationships that could be construed as a potential conflict of interest.
